# Artificial Intelligence and Bioengineering Approaches for Antimicrobial Resistance Prediction

**DOI:** 10.3390/medicina62091651

**Published:** 2026-08-28

**Authors:** Oana Frandeș, Leonard Azamfirei, Oana Elena Branea, Sergiu Ioan Frandeș, Alexandra Elena Lazăr

**Affiliations:** 1 Doctoral School of Medicine and Pharmacy, George Emil Palade University of Medicine, Pharmacy, Science and Technology of Târgu Mureș, 540142 Targu Mures, Romania; oana.frandes@umfst.ro (O.F.); sergiu.frandes@umfst.ro (S.I.F.); 2 Department of Anesthesiology and Intensive Care Medicine, George Emil Palade University of Medicine, Pharmacy, Science and Technology of Târgu Mureș, 540103 Targu Mures, Romania; oana.branea@umfst.ro (O.E.B.); alexandra.lazar@umfst.ro (A.E.L.)

**Keywords:** artificial intelligence, antimicrobial resistance, machine learning, antimicrobial stewardship, intensive care unit, clinical decision support, bioengineering, MALDI-TOF MS, whole-genome sequencing

## Abstract

*Background and Objectives:* Antimicrobial resistance (AMR) is a major challenge, particularly in intensive care units, where broad-spectrum therapy is often initiated before microbiological confirmation. Artificial intelligence (AI) may improve AMR prediction, but its clinical value depends on integration with bioengineering-enabled digital microbiology. This narrative review examines how AI, bioengineering platforms and digital microbiology can support AMR prediction, clinical decision support and antimicrobial stewardship across the sample-to-decision pipeline. *Materials and Methods:* A targeted narrative review was conducted using PubMed/MEDLINE and Google Scholar. Publications from 2020 onward were prioritized, while earlier seminal studies, methodological frameworks and regulatory documents were included when relevant. Evidence was synthesized across AI-based resistance prediction, antimicrobial stewardship, digital microbiology and bioengineering technologies. *Results:* AI and machine-learning approaches showed promising performance in patient-level resistance prediction, pathogen-level susceptibility prediction and antimicrobial stewardship. For example, model discrimination reached an AUROC of 0.936 for carbapenem-resistant *Klebsiella pneumoniae* prediction, while model-guided empirical therapy in *Enterobacterales* bloodstream infections could have increased active beta-lactam therapy from 70% to 79%. However, most evidence remains retrospective and single-centre, with limited external or prospective validation. *Conclusions:* AI has considerable potential to support AMR prediction and antimicrobial stewardship, but current evidence primarily demonstrates technical feasibility rather than established clinical effectiveness. Broader implementation will require rigorous validation, integration into clinical workflows, continuous monitoring and demonstration of clinical benefit.

## 1. Introduction

Antimicrobial therapy for severe infections is frequently initiated before the causative pathogen and its antimicrobial susceptibility profile are known. Although this empirical approach is often lifesaving, it also exposes many patients to unnecessary broad-spectrum antibiotics, increasing selective antimicrobial pressure and contributing to the emergence and spread of antimicrobial resistance (AMR). Inappropriate antimicrobial use, together with transmission of resistant microorganisms, remains one of the principal drivers of AMR, particularly in healthcare settings and intensive care units (ICUs), where critically ill patients require immediate treatment despite considerable diagnostic uncertainty [1,2,3].

Conventional microbiological diagnostics cannot always provide information rapidly enough to guide early targeted therapy. Microbiological identification and antimicrobial susceptibility testing (AST) generally require 24–72 h, during which clinicians must make therapeutic decisions despite uncertainty regarding both the causative pathogen and its resistance profile. Consequently, broad-spectrum empirical therapy is frequently prescribed, increasing selective pressure and predisposing patients to complications such as *Clostridioides difficile* infection [1,4,5].

Artificial intelligence (AI), particularly machine learning (ML), has emerged as a promising tool for integrating heterogeneous clinical, microbiological and laboratory data into clinically meaningful predictions. By identifying complex patterns beyond conventional statistical approaches, AI has the potential to reduce diagnostic uncertainty and support more timely and individualized antimicrobial prescribing [1,2].

AI should not be considered just a predictive algorithm, but rather as one part of a broader bioengineering and digital microbiology ecosystem [6,7]. Modern diagnostic technologies, including whole-genome sequencing (WGS), metagenomic next-generation sequencing (mNGS), matrix-assisted laser desorption/ionization time-of-flight mass spectrometry (MALDI-TOF MS), automated AST, biosensors, microfluidic platforms and electronic health records (EHRs), transform biological samples into standardized digital data that can be integrated and analyzed by AI models [6,8]. The successful implementation of AI therefore depends not only on algorithm development, but also on robust diagnostic platforms, reliable digital infrastructure and effective integration into routine clinical workflows [1,2].

The aim of this narrative review is to analyze how artificial intelligence and bioengineering-enabled digital microbiology platforms can be integrated to support antimicrobial resistance prediction and clinical decision support. Recent reviews have examined the role of artificial intelligence and machine learning in antimicrobial resistance prediction, antimicrobial stewardship and optimization of antimicrobial prescribing [1,2,9]. The novelty of this narrative review lies in its integration of AI-based antimicrobial resistance prediction within a broader bioengineering-enabled digital microbiology framework. Rather than focusing primarily on predictive algorithms or individual AI applications, the review follows the sample-to-decision pipeline, from biological sample acquisition and diagnostic platforms to structured digital data, AI-based prediction and clinician-supervised decision support. AI applications are examined at three complementary levels: patient-level prediction of multidrug-resistant microorganism (MDRO) colonization or infection, pathogen-level prediction of antimicrobial susceptibility, and unit-level assessment of epidemiological risk and resistance dynamics. The review also addresses the technical, biological and organizational requirements necessary to translate predictive performance into real-world clinical implementation. Although antimicrobial resistance encompasses bacterial, fungal and viral pathogens, the present review focuses specifically on bacterial antimicrobial resistance. Antifungal and antiviral resistance, including emerging challenges such as resistance in *Candida auris*, are beyond the scope of this review.

## 2. Materials and Methods

This narrative literature review summarizes current evidence regarding the application of artificial intelligence (AI) for antimicrobial resistance (AMR) prediction and antimicrobial stewardship, with particular emphasis on intensive care settings. The review also explores the contribution of bioengineering, digital microbiology and rapid diagnostic technologies to AI-assisted clinical decision-making.

A targeted literature search was conducted using PubMed/MEDLINE and Google Scholar between 11 and 28 June 2026. Targeted supplementary searches were subsequently performed to identify additional evidence and methodological, regulatory and technological references, with the final search update completed on 20 August 2026. Priority was given to publications published from 2020 onward, reflecting the rapid development of AI, machine learning and digital diagnostic technologies in AMR research. Earlier publications were considered when they represented seminal studies, methodological frameworks, regulatory documents or key technological references relevant to the scope of the review.

Searches were performed across predefined thematic domains corresponding to the major sections of the review. Search terms combined concepts related to AI and machine learning with AMR, antimicrobial stewardship, intensive care, electronic health records, microbiology, whole-genome sequencing, metagenomic next-generation sequencing, MALDI-TOF mass spectrometry, rapid diagnostic technologies, bioengineering, digital microbiology, biosensors, microfluidics and clinical decision support. The principal database-specific search strategies are provided in Supplementary Table S1.

Original research articles, systematic and narrative reviews, clinical and methodological guidelines, and relevant regulatory documents published in English were considered. Publications were selected according to their relevance to the objectives of the review, with emphasis on AI-based AMR prediction, antimicrobial susceptibility prediction, antimicrobial stewardship, digital microbiology, bioengineering platforms and clinical decision support. Conference abstracts, duplicate publications and studies not directly relevant to the scope of the review were excluded. Reference lists of selected publications were additionally screened to identify further relevant sources.

Literature identification and selection were performed by one author using a topic-driven approach. Because this was a narrative review and searches were conducted separately across multiple predefined thematic domains, records were not prospectively tracked through a formal systematic screening workflow.

## 3. Clinical Need for AI-Enabled AMR Prediction

The ICU represents one of the most challenging environments for antimicrobial stewardship. It has often been described as an amplification chamber for AMR because critically ill patients are frequently exposed to broad-spectrum antimicrobials, invasive procedures and prolonged hospitalization [1,2]. At the same time, the ICU is one of the richest sources of clinical, microbiological and biological data generated during routine patient care. Continuous monitoring provides information on vital signs, laboratory investigations, microbiological cultures, antimicrobial susceptibility testing, previous antimicrobial exposure, invasive devices, patient transfers, colonization status and, increasingly, molecular diagnostic results [6,9]. These heterogeneous data make the ICU particularly suitable for AI applications, provided that they are standardized and integrated into digital healthcare systems [6,9,10].

### 3.1. ICU as an Amplification Chamber

ICUs represent some of the healthcare environments most affected by AMR. This phenomenon is driven by the high burden of comorbidities and vulnerability of critically ill patients, the extensive use of antimicrobial therapies, and the presence of multiple entry points for microorganisms. Together, these factors contribute to the emergence and transmission of MDROs. In the study conducted by Alparslan et al., diabetes mellitus, cerebrovascular disease and immunosuppressive therapy were significantly more frequent among patients with carbapenem-resistant *Klebsiella pneumoniae* infection [11]. Furthermore, prolonged hospitalization and longer ICU stay were identified as important predictors of carbapenem resistance, highlighting the close relationship between critical illness and the risk of acquiring MDROs [11,12]. As a result, ICUs are often considered amplification chambers where resistant pathogens can be selected, maintained and disseminated.

One of the most important factors contributing to this phenomenon is the high vulnerability of critically ill patients. Severe diseases, immune dysfunction, prolonged hospitalization and multiple exposures to healthcare interventions increase both colonization and infection with resistant microorganisms [12,13,14].

Another important mechanism is the high number of invasive medical devices used in ICUs. Central venous catheters, urinary catheters, mechanical ventilation, parenteral nutrition, and other life-support devices modify natural barriers and provide potential entry points for pathogens. This circumstance increases the risk of healthcare-associated infections. Among invasive interventions, prolonged mechanical ventilation was significantly associated with carbapenem-resistant *Klebsiella pneumoniae* infection. These findings illustrate how invasive procedures may contribute to the disruption of natural host barriers and increase susceptibility to colonization and infection with resistant microorganisms [11].

High antimicrobial consumption also contributes to the development of resistance. Broad-spectrum antibiotics are frequently prescribed empirically before microbiological results become available because of the severity of illness and the need for rapid therapeutic intervention. Ziaian et al. reported in their study that 96% of ICU patients received broad-spectrum antimicrobial therapy, with a median duration of therapy of nearly 30 days and virtually no antibiotic-free days during hospitalization. In addition, more than one-third of antibiotic prescriptions were considered inappropriate [12]. Such extensive antimicrobial exposure increases the selective pressure, leading to high survival and dissemination of MDROs, particularly resistant Gram-negative bacteria.

ICUs are defined by complex transmission networks that include patients, healthcare workers, medical equipment, and the hospital environment. El Arab et al. report that ICUs experience some of the highest rates of healthcare-associated infections, primarily due to prolonged hospitalization, extensive use of invasive devices, and frequent patient contact. These factors promote the transmission of MDROs and support the persistence of resistant strains in the unit [14]. Therefore, AMR in ICUs should be seen as a dynamic phenomenon in which host vulnerability, invasive procedures, antimicrobial pressure, and pathogen transmission are interrelated.

### 3.2. The Uncertainty Spiral and Its Clinical Consequences

The management of suspected infections in critically ill patients requires the initiation of antimicrobial therapy; however, information regarding the causative pathogen and its antimicrobial susceptibility is often unavailable at the time of ICU admission. Furthermore, delays in treatment initiation may lead to increased morbidity and mortality. In this context, empirical broad-spectrum antimicrobial therapy is commonly prescribed in order to maximize the probability of adequate initial coverage [1,3].

Therapeutic inertia further contributes to this problem. Even after microbiological results become available, de-escalation is often delayed due to clinical deterioration of the patient, lack of confidence in microbiological findings, or simply oversight. As a result, broad-spectrum antimicrobial therapy may be continued for longer than necessary, increasing selective pressure and promoting the emergence of resistant microorganisms. As a result, exposure to broad-spectrum antibiotics is often prolonged beyond what is clinically necessary [1].

These events create what may be described as an “uncertainty spiral.” Diagnostic uncertainty promotes empirical broad-spectrum prescribing, which increases antimicrobial pressure and accelerates the emergence of resistant microorganisms. Rising resistance subsequently makes future empirical treatment decisions even more difficult, generating additional uncertainty and perpetuating the cycle.

Reducing clinical uncertainty is a critical target for future antimicrobial stewardship interventions and provides an important rationale for integrating AI-based decision support systems into clinical practice [1,2,3].

### 3.3. Limitations of Current Stewardship and Surveillance Frameworks

At the international level, surveillance and antimicrobial stewardship efforts are supported by major initiatives such as the World Health Organization Global Antimicrobial Resistance and Use Surveillance System (WHO GLASS), the European Centre for Disease Prevention and Control European Antimicrobial Resistance Surveillance Network (ECDC EARS-Net), and the U.S. Centers for Disease Control and Prevention National Healthcare Safety Network (CDC NHSN). These surveillance systems provide standardized epidemiological and microbiological data that can support the monitoring of resistance trends and inform public health and infection-control strategies [2,15].

Current stewardship programs often rely on human data review, delayed microbiological results, and retrospective analysis of resistance trends. While these methods are valuable for monitoring AMR, they provide limited support for real-time clinical decision-making and have little ability to predict future resistance patterns or transmission events. These limitations highlight the need for more proactive and data-driven approaches, creating an opportunity to integrate AI into antimicrobial stewardship and surveillance [2,9,16].

## 4. Digital Microbiology Data Streams for AI-Based AMR Prediction

The use of AI models is becoming increasingly common; however, their performance depends primarily on the quantity and quality of the data used. These models cannot generate clinically useful results when relying on a single type of data. For this reason, the most valuable outcomes are achieved through the combined use of clinical, microbiological and genomic information [1,9].

### 4.1. Clinical and EHR Data

Clinical data, demographic characteristics and patient history represent some of the most accessible and widely available sources of information that can be used for the development and training of AI models. As these data are routinely collected, they provide demographic characteristics such as age, sex and place of residence, as well as information regarding patient comorbidities, previous hospitalizations, treatment history and current clinical status [9,17,18].

EHRs represent more than a passive repository of clinical information. They constitute the digital infrastructure through which AI models can integrate heterogeneous patient data, including previous hospitalizations, prior antimicrobial exposure, microbiological history, colonization status, invasive devices, disease severity scores and laboratory results [9].

The integration of demographic characteristics, previous healthcare exposure, treatment history and infection-related variables enables AI models to identify patterns associated with AMR. By integrating these heterogeneous data sources, AI models may support early risk stratification and contribute to individualized antimicrobial treatment decisions before definitive microbiological results become available [9].

### 4.2. Microbiological and Epidemiological Data

Microbiological data, although usually available 24–72 h after sample collection, provide some of the most valuable information regarding AMR and are used by nearly all AI models developed to predict resistance. These data include culture results, antimicrobial susceptibility testing, previous colonization with MDROs, and local resistance patterns observed at the level of the ICU or healthcare institution [9,19,20].

Epidemiological information plays an important role in AMR prediction. Local antibiograms, resistance trends, and surveillance data may reflect the prevalence of specific resistant pathogens within a healthcare setting. Because resistance patterns vary between hospitals and geographical regions, the integration of epidemiological information allows predictive models to account for the local context in which infections occur [9,21,22,23].

The combination of clinical, microbiological and epidemiological data provides a better representation of the factors contributing to AMR. By integrating these complementary sources of information, AI models may improve risk prediction, support antimicrobial stewardship initiatives and facilitate more informed therapeutic decisions [9,22,23].

### 4.3. Genomic, Proteomic and Phenotypic Data

Genomic, proteomic and phenotypic data provide complementary microorganism-specific information for AI-based AMR prediction. Genomic data may include resistance-associated genes, mutations and other sequence-derived features, whereas proteomic and phenotypic data may consist of mass spectra, growth characteristics, MIC values or other measurable responses to antimicrobial exposure. These heterogeneous data can be transformed into structured digital inputs for machine-learning models and may support the prediction of antimicrobial susceptibility before conventional testing results become fully available [9,24,25,26].

The clinical value of these data depends on the characteristics and performance of the platforms that generate them. MALDI-TOF MS, WGS, mNGS, automated and microfluidic AST, and emerging biosensor-based technologies differ substantially in the biological signals they measure, the type of digital data generated and their current level of clinical readiness. These bioengineering platforms and their relevance to AI-enabled AMR prediction are discussed in the following section [1,9,27,28,29].

### 4.4. Local Antibiograms and Surveillance Data

Local antibiograms remain one of the most widely used tools for guiding empirical antimicrobial therapy and antimicrobial stewardship. By summarizing cumulative antimicrobial susceptibility data at the hospital or ICU level, they provide clinicians with an overview of local resistance patterns and support empirical treatment decisions before patient-specific microbiological results become available. However, conventional antibiograms are inherently retrospective and provide only a static representation of antimicrobial susceptibility, without accounting for temporal variations in resistance or ongoing changes in local epidemiology [6,9].

The digitalization of clinical microbiology has considerably expanded the range of epidemiological data available for antimicrobial surveillance. Information derived from laboratory information systems (LISs), electronic health records (EHRs), AST platforms and regional surveillance programs can be integrated to generate dynamic representations of local resistance epidemiology. These data streams allow continuous monitoring of pathogen distribution, antimicrobial susceptibility patterns and temporal resistance trends, providing a broader epidemiological context for AI-based prediction and clinical decision support [2,6].

## 5. Bioengineering Platforms for AI-Enabled AMR Prediction

### 5.1. MALDI-TOF MS and Proteomic Data

Rapid diagnostic technologies have significantly improved the diagnosis of infectious diseases by reducing the time required for pathogen identification and antimicrobial susceptibility testing. Among these technologies, MALDI-TOF MS has become a cornerstone of modern clinical microbiology. Rather than analyzing the instrument itself, AI models analyze the proteomic spectra produced by MALDI-TOF MS, identifying spectral patterns associated with specific antimicrobial resistance phenotypes. In parallel, the transition from conventional microbiology laboratories to digital microbiology platforms has enabled the generation of standardized, high-quality microbiological data through laboratory automation, digital imaging and integrated laboratory information systems. These technologies not only improve laboratory workflow and turnaround time but also provide structured digital information that can be integrated with AI models to support AMR prediction and clinical decision-making [6,7].

MALDI-TOF MS has become one of the most important technologies implemented in routine clinical microbiology laboratories. In addition to rapid microorganism identification, recent advances in machine learning have shown that MALDI-TOF MS can also be used to predict AMR by identifying proteomic patterns associated with specific resistance phenotypes. Compared with conventional susceptibility testing, this approach may provide clinically relevant information earlier and facilitate timely optimization of antimicrobial therapy. However, model performance depends on spectrum quality, sample preparation, instrument type, local reference databases, inter-laboratory variability and local microbial epidemiology. Consequently, models developed in one laboratory cannot be assumed to perform equally well in different healthcare settings without external validation [10,30].

### 5.2. WGS, mNGS and Genomic Data

Molecular and genomic technologies have considerably expanded the range of information available for AMR prediction. Unlike conventional microbiological methods, sequencing-based approaches provide detailed information on resistance-associated genes, chromosomal mutations, plasmids and clonal relationships, allowing a better understanding of the molecular mechanisms involved in AMR. These data can be integrated into AI models to identify complex genomic patterns associated with specific resistance phenotypes and to support epidemiological surveillance [8,31].

WGS enables comprehensive analysis of the entire bacterial genome and facilitates the identification of known resistance genes, novel mutations and mobile genetic elements involved in AMR. In addition to pathogen characterization, WGS provides valuable information for outbreak investigation, strain typing and surveillance of resistance dissemination. When combined with machine learning, genomic data can be used to predict antimicrobial susceptibility directly from genomic features. However, the clinical implementation of WGS remains limited by sequencing costs, bioinformatic complexity and the imperfect correlation between genotype and phenotype. The presence of a resistance gene does not necessarily result in phenotypic resistance, whereas the absence of known resistance genes does not exclude resistance mediated by novel mutations, regulatory mechanisms or other complex biological processes [8,31].

mNGS further expands these capabilities by enabling the direct detection of pathogens and resistance-associated genes from clinical specimens without the need for prior culture. This approach may shorten time to microbiological diagnosis and improve pathogen identification, particularly in polymicrobial infections or in patients receiving antimicrobial therapy before sample collection. Despite these advantages, several challenges remain, including host DNA contamination, complex data analysis, high costs and the difficulty of correlating detected resistance genes with the expressed resistance phenotype. AI-based analytical tools may facilitate the interpretation of these large and complex datasets. However, further clinical validation is required before implementation in routine clinical practice [8].

### 5.3. Automated and Microfluidic AST

Automated AST systems and emerging microfluidic platforms generate phenotypic data that reflect the biological response of microorganisms to antimicrobial exposure. Depending on the platform, susceptibility can be assessed through changes in bacterial growth, metabolic activity, turbidity, fluorescence, microscopic imaging or other time-dependent phenotypic signals, which are subsequently converted into MIC values or susceptibility categories [32]. Conventional automated systems, including VITEK 2, BD Phoenix, MicroScan and Sensititre, remain widely used in clinical microbiology laboratories, whereas newer rapid platforms aim to shorten the time required for phenotypic susceptibility determination through automated imaging, kinetic growth analysis and microscale testing approaches [32].

Microfluidic AST represents a particularly relevant bioengineering approach because it enables the analysis of bacterial responses to antimicrobial exposure in small-volume environments while generating continuous or time-dependent digital phenotypic data. These platforms can combine microfluidic sample handling with real-time growth monitoring and image-based analysis, allowing susceptibility and MIC determination within a substantially shorter timeframe than conventional workflows [32,33]. For example, the QuickMIC platform uses a microfluidic system to generate rapid phenotypic susceptibility data and has been evaluated in routine clinical practice for Gram-negative bacteremia. In this study, actionable MIC results were obtained within 2–4 h, with 99.02% concordance with reference methods across 816 antibiotic–microorganism combinations [34].

The clinical value of these platforms extends beyond faster AST results because the phenotypic signals they generate can be incorporated into automated analytical pipelines and potentially combined with AI-based models. However, rapid phenotypic AST technologies remain heterogeneous with respect to their measurement principles, antimicrobial panels, sample requirements and level of clinical validation. Their broader implementation therefore depends on analytical standardization, agreement with reference AST methods, integration into laboratory workflows and demonstration of clinical benefit beyond improved turnaround time [32,34].

### 5.4. Biosensor-Based AMR Detection

Biosensor-based platforms represent an emerging bioengineering approach for AMR detection by converting biological recognition events into measurable signals. Depending on their design, biosensors may detect bacterial biomarkers, resistance-associated proteins or nucleic acid targets, including antimicrobial resistance genes. These interactions can generate electrochemical, optical or other measurable outputs. For example, electrochemical biosensors convert interactions between a biological recognition element and its target into signals such as changes in current, potential or impedance, which can subsequently be transformed into structured digital data for automated analysis [35,36].

The digital signals generated by biosensors may provide a potential input for AI- and machine-learning-based analytical pipelines. Depending on the sensing platform, these data may include electrochemical measurements, spectral patterns or image-based signals, which can be analyzed to identify patterns associated with pathogens or resistance phenotypes [37]. In this context, biosensors may complement conventional microbiological methods by enabling rapid detection of resistance-associated targets or early phenotypic responses directly from biological samples [35,36].

However, most biosensor-based approaches remain emerging technologies and their translation into routine clinical microbiology is still limited. Important barriers include analytical standardization, signal reproducibility, performance in complex clinical samples, integration into existing diagnostic workflows and the need for further clinical validation and cost-effectiveness assessment [35,36]. Therefore, although biosensors may expand the range of biological signals available for AI-based AMR prediction, their clinical utility will depend not only on analytical performance but also on successful validation and integration into real-world microbiology and clinical decision-support systems.

The main characteristics, data outputs, practical requirements and current clinical readiness of these bioengineering platforms are summarized in Table 1.

### 5.5. LIS, EHR and Clinical Decision-Support Integration

Laboratory information systems (LISs) and electronic health records (EHRs) constitute the digital infrastructure that enables the integration of microbiological, molecular and clinical data into unified datasets suitable for AI analysis. While LISs manage laboratory-generated information such as culture results, AST, MALDI-TOF MS spectra and molecular diagnostic findings, EHRs provide complementary clinical information, including patient demographics, comorbidities, previous antimicrobial exposure, laboratory parameters and hospitalization history [1,6,7].

The integration of LIS and EHR data allows AI models to analyze heterogeneous information within a single analytical framework, supporting antimicrobial resistance prediction and clinical decision-making. Unlike individual diagnostic platforms, which generate specific microbiological or molecular signals, digital health systems provide the interoperability required to combine these complementary data streams into clinically meaningful datasets. Consequently, the performance of AI models depends not only on algorithm design but also on data quality, standardization, interoperability and the completeness of integrated clinical and microbiological information [1,2,7].

**Figure 1:**
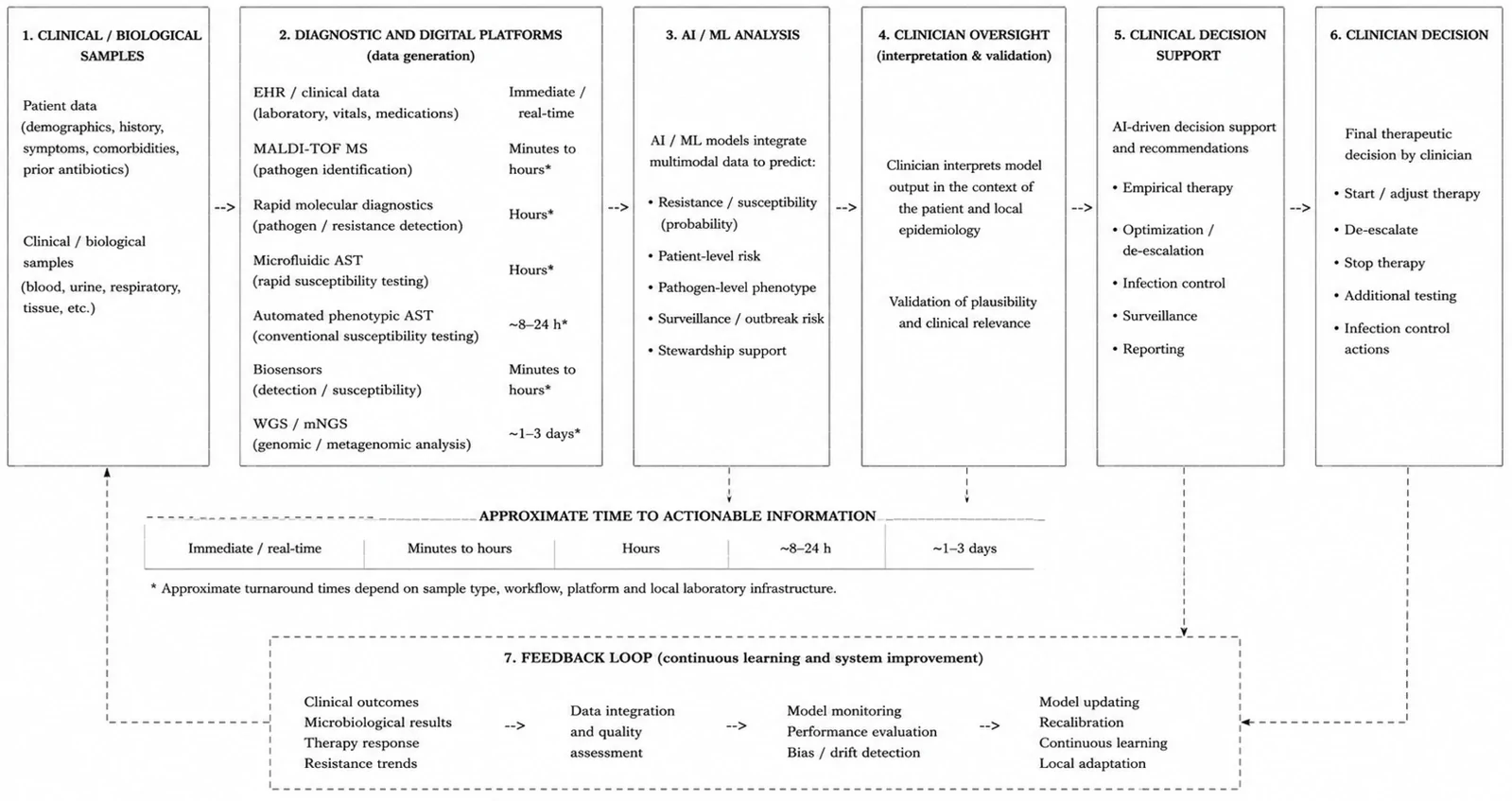
Sample-to-decision pipeline illustrating the integration of bioengineering diagnostic platforms, structured digital microbiology data and artificial intelligence to support antimicrobial resistance prediction and antimicrobial stewardship. Approximate turnaround times indicate the potential time to actionable information and may vary according to sample type, workflow and local infrastructure. Clinician oversight is required to interpret AI-generated outputs before therapeutic decisions are made. Continuous feedback from clinical implementation enables model monitoring and recalibration.

Despite these advantages, several technical challenges remain. Differences in data formats, incomplete documentation, heterogeneous coding systems and limited interoperability between healthcare information systems may reduce data quality and restrict the generalizability of AI models. Therefore, standardized digital infrastructures and continuous data quality assurance are essential prerequisites for reliable AI-supported clinical decision-making [1,2]. The overall pathway from sample collection to AI-assisted clinical decision-making is illustrated in Figure 1.

## 6. AI Applications in AMR Prediction and Decision Support

AI applications for antimicrobial resistance prediction can be broadly categorized into three complementary levels according to the clinical question they address. Patient-level prediction estimates the probability that an individual patient is colonized or infected with MDROs before microbiological confirmation becomes available. Pathogen-level prediction focuses on determining whether a bacterial isolate is susceptible or resistant to a specific antimicrobial agent using microbiological, phenotypic, proteomic or genomic data. Finally, unit-level prediction integrates surveillance and epidemiological information to estimate colonization pressure, detect transmission dynamics and support infection prevention strategies within healthcare units. Together, these complementary prediction levels support antimicrobial stewardship by providing decision support from the individual patient to the institutional level.

It is important to distinguish between different levels of AI-assisted AMR analysis. Detection of resistance-associated genes or mutations identifies known genetic determinants, whereas resistance-mechanism identification aims to characterize the biological processes underlying reduced antimicrobial susceptibility. Phenotypic AMR prediction represents a further step, integrating genomic, proteomic, spectral or clinical data to infer the observed susceptibility phenotype or minimum inhibitory concentration. Finally, clinical decision-support models operate at a different level by combining resistance predictions with patient-specific and contextual information to support empirical antimicrobial selection or treatment optimization. These applications are therefore complementary but should not be considered interchangeable, as the detection of a resistance determinant does not necessarily translate directly into phenotypic resistance or an appropriate patient-specific treatment recommendation [38].

### 6.1. Patient-Level Resistance Risk Prediction

The selection of empirical antimicrobial therapy in critically ill patients is frequently complicated by the absence of microbiological results at the time treatment decisions are made. Broad-spectrum antibiotics are often prescribed to maximize the probability of adequate initial coverage. AI models have emerged as promising tools for identifying patients at increased risk of infection with MDROs [1,2].

AI-based patient-level resistance risk prediction primarily relies on clinical and electronic health record data rather than on molecular diagnostic technologies. These models integrate information such as previous microbiological results, prior antimicrobial exposure, comorbidities, hospitalization history, invasive devices and laboratory findings to estimate the probability that a patient is colonized or infected with MDROs before microbiological confirmation becomes available. By combining these heterogeneous clinical data, AI algorithms can identify patterns associated with AMR that may not be apparent during routine clinical assessment. Consequently, these models may support early risk stratification and guide empirical antimicrobial therapy [9,12,39].

Several studies have demonstrated the potential of patient-level AMR prediction models. Alparslan et al. developed an XGBoost model for the prediction of carbapenem-resistant *Klebsiella pneumoniae* infections in critically ill patients. The model achieved an accuracy of 83% and a sensitivity of 88%, highlighting the ability of ML algorithms to identify patients at increased risk of resistant infections before microbiological confirmation [11].

Nguyen et al. and Davis et al. demonstrated the potential of machine-learning approaches to infer antimicrobial susceptibility from genomic data, whereas McGuire et al. developed an XGBoost model using routinely available clinical variables to predict carbapenem-resistant infections at the time of culture collection, achieving an AUROC of 0.846 [17,18,19].

More directly relevant to empirical therapy in critically ill patients, Feretzakis et al. evaluated machine-learning models in 345 ICU patients and showed that routinely available clinical and microbiological information could be used to support empirical antibiotic selection, with reported AUROCs of approximately 0.70–0.73 (Feretzakis et al.). Similarly, Oonsivilai et al. demonstrated that locally available patient data could be used to generate individualized susceptibility predictions in a resource-limited paediatric hospital setting; a random forest model achieved AUROCs ranging from 0.71 to 0.85 for different empirical treatment-related outcomes [21].

Vazquez-Guillamet et al. further illustrated the clinical relevance of patient-level resistance prediction by developing regression and decision-tree models to stratify septic patients with Gram-negative bloodstream infections according to their risk of resistance to piperacillin–tazobactam, cefepime and meropenem. Using simple clinical variables, including previous antibiotic exposure and healthcare-associated risk factors, the models identified patient groups with different probabilities of resistance, providing a potential framework for more individualized empirical therapy [40].

Ziaian et al. developed ML models to predict MDR and extensively drug-resistant (XDR) infections in ICU patients using routinely available clinical and microbiological data. Their findings demonstrated that factors such as prolonged hospitalization, previous antimicrobial exposure and severity of illness contributed significantly to AMR prediction, supporting the idea of AI-assisted risk stratification in critically ill populations [12].

Kim et al. developed a multitask ML model using data from nearly 60,000 patients across three hospitals to predict resistance to multiple antibiotic classes simultaneously, highlighting the value of longitudinal microbiological history for individualized empirical treatment [39]. In addition, Yuan et al. provided one of the most clinically relevant evaluations by directly comparing model predictions with clinicians’ empirical prescribing decisions in 4709 Enterobacterales bloodstream infection episodes. Although model discrimination was modest (AUROC 0.680–0.737 without species identification and up to 0.827 after species information became available), model-guided treatment could have increased the proportion of patients receiving an active beta-lactam from 70% to 79%, while reducing under treatment from 30% to 21%. These findings illustrate that clinical usefulness may not be fully captured by discrimination metrics alone and that comparison with actual clinician decision-making represents an important step toward evaluating the practical value of AI-assisted AMR prediction [41].

By identifying patients at increased risk of resistant infections before culture results become available, AI-based prediction models may support more appropriate empirical antimicrobial therapy, reduce unnecessary exposure to broad-spectrum antibiotics and strengthen antimicrobial stewardship efforts. Although further external validation is required before widespread implementation, these models represent one of the most clinically applicable uses of AI in AMR management [1,2,3].

### 6.2. Pathogen-Level Susceptibility Prediction

Rapid antimicrobial susceptibility prediction is closely linked to advances in bioengineering and rapid diagnostic technologies. Platforms such as MALDI-TOF MS, WGS, mNGS, automated AST, microfluidic systems and biosensors produce molecular, proteomic or phenotypic data that can be analyzed using AI algorithms. By interpreting these biological signals, AI models may predict antimicrobial susceptibility earlier than conventional microbiological methods and support more timely treatment decisions [8,10,31,33].

The identification of antimicrobial susceptibility remains one of the most important challenges in the management of severe infections. Conventional culture-based susceptibility testing usually requires 24–72 h after microbiological samples are collected, but empirical therapy is initiated immediately. Delays in identifying resistant pathogens may contribute to inappropriate antimicrobial therapy and clinical deterioration of the patient [1,2].

Unlike patient-level resistance prediction, antimicrobial susceptibility prediction focuses on the pathogen rather than the patient. Its objective is to estimate whether an identified microorganism is susceptible or resistant to specific antimicrobial agents. AI models developed by clinicians and researchers may be capable of predicting antimicrobial susceptibility using genomic and microbiological data before conventional susceptibility testing is reported. WGS, mNGS and MALDI-TOF MS, produce large amounts of data that can be analyzed using ML algorithms to identify resistance-associated patterns and predict susceptibility profiles [2,9].

One of the most promising applications involves the combination of ML algorithms with MALDI-TOF MS. Beyond rapid microorganism identification, MALDI-TOF MS can be analyzed using AI models to detect resistance-associated patterns. Wang et al. developed ML models for the rapid detection of carbapenem-resistant *Klebsiella pneumoniae* (CRKP) using MALDI-TOF data. Among the evaluated algorithms, the SVM-K model achieved the best performance, with an accuracy of 91%, sensitivity of 89%, specificity of 94% and an AUC of 0.936, demonstrating the potential of AI-assisted AMR prediction in clinically important MDROs [29].

Similar models have been applied to other resistant microorganisms. Wang et al. developed a deep learning model based on MALDI-TOF MS for the prediction of vancomycin-resistant *Enterococcus faecium* (VREfm). Using external validation, the model achieved an AUROC of 0.887, supporting the feasibility of rapid susceptibility prediction directly from routinely generated microbiological data [28]. Earlier studies also demonstrated the ability of ML algorithms to identify heterogeneous vancomycin-intermediate *Staphylococcus aureus* (hVISA) isolates using MALDI-TOF MS, with the best-performing SVM model achieving an AUC of 0.79, sensitivity of 0.77 and specificity of 0.81 on nested cross-validation [27]. The variable performance of these models can be explained by two factors. The first factor is that microorganisms have different resistance mechanisms (carbapenemase production in CRKP, clonal diversity of VREfm with 24 sequence types identified, and a polygenic heterogeneous phenotype in hVISA). The second factor is the use of validation methods with different degrees of rigour (nested cross-validation in the hVISA study versus more permissive approaches in the other studies) [27,28,29].

Another method is represented by mNGS. Li et al. developed an mNGS-based ML platform for antimicrobial susceptibility prediction in five major ESKAPEE pathogens, including *Acinetobacter baumannii, Klebsiella pneumoniae, Escherichia coli*, *Pseudomonas aeruginosa* and *Staphylococcus aureus*. The model achieved an overall accuracy of 93.84% while significantly reducing reporting time compared with conventional susceptibility testing (1.12 ± 0.33 versus 2.81 ± 0.57 days). Furthermore, the authors estimated that the system could allow earlier optimization of antimicrobial therapy in approximately one-third of culture-positive patients. However, accuracy for *Pseudomonas aeruginosa* was only 84.38%, while the predictable ratio for colistin reached as low as 6.1%, reflecting the complex resistance mechanisms of *P. aeruginosa*—including efflux pumps and low-permeability outer membrane barriers—which remain difficult to capture through genomic analysis alone. The authors also report one case of incorrect resistance gene attribution, from *Escherichia coli* to *Enterococcus faecium*, underscoring the need for human oversight in the clinical interpretation of model outputs [42].

These findings support the idea that AI-assisted susceptibility prediction may provide clinically relevant information hours or even days before conventional susceptibility testing becomes available. By shortening the interval between pathogen detection and targeted treatment, AI-based susceptibility prediction has the potential to improve antimicrobial selection, reduce unnecessary exposure to broad-spectrum antibiotics and strengthen antimicrobial stewardship programs [1,2].

### 6.3. Unit-Level Surveillance and Colonization Pressure

Colonization pressure refers to the epidemiological burden created by the presence of patients colonized or infected with MDROs within a healthcare unit over a defined period. It reflects not only the number of affected patients but also the potential exposure of other patients to resistant organisms. Increased colonization pressure has been associated with a higher risk of MDRO transmission and healthcare-associated infection, making it a relevant parameter for infection prevention and epidemiological surveillance [1,14].

Conventional surveillance systems generally provide retrospective summaries of microbiological results and resistance patterns. However, the increasing availability of longitudinal data from microbiology laboratories, electronic health records and antimicrobial use databases creates opportunities for more dynamic assessment of local epidemiology. AI-based analytical approaches may integrate these heterogeneous data over time to identify changes in pathogen distribution, resistance patterns and other signals potentially associated with increased transmission risk [1,9,14].

At the unit level, these models may complement conventional surveillance by moving beyond static descriptions of resistance epidemiology toward dynamic risk assessment. For example, continuously updated data may allow the identification of temporal trends, unexpected increases in the occurrence of specific resistant organisms or changes in the epidemiological profile of a healthcare unit. Such information could support earlier investigation of potential outbreaks and help prioritize infection prevention measures or targeted surveillance activities [1,14].

An important advantage of AI-based unit-level surveillance is the potential integration of multiple data streams into a continuously updated epidemiological framework. Rather than replacing conventional infection control procedures, these systems could function as an additional decision-support layer, helping infection prevention and antimicrobial stewardship teams interpret complex longitudinal data. However, evidence supporting the real-world clinical impact of AI-based unit-level surveillance remains limited. Further prospective studies are needed to determine whether these approaches can improve outbreak detection, reduce MDRO transmission or meaningfully influence infection prevention and stewardship outcomes [3].

### 6.4. Antimicrobial Stewardship and De-Escalation Support

AI may support antimicrobial de-escalation by integrating clinical information, microbiological results, antimicrobial susceptibility testing and previous antimicrobial therapy. These systems can identify patients who may benefit from treatment optimization and highlight potential opportunities for antimicrobial de-escalation. However, treatment decisions should always be reviewed and validated by the treating physician and the antimicrobial stewardship team before therapy is modified [16,43].

Antimicrobial de-escalation represents an important component of antimicrobial stewardship programs, aiming to reduce unnecessary exposure to broad-spectrum antibiotics while maintaining adequate treatment of infections. In critically ill patients, de-escalation commonly involves narrowing antimicrobial spectrum, discontinuing unnecessary agents, or switching from empirical broad-spectrum therapy to targeted treatment once additional microbiological information becomes available. However, these decisions are frequently influenced by clinical uncertainty and may be delayed despite the availability of relevant clinical and microbiological data [1,44].

Clinical decision support systems represent the final step in the AI workflow by translating resistance risk estimates and antimicrobial susceptibility predictions into clinically actionable recommendations. Rather than replacing clinical judgement, these systems assist clinicians in optimizing empirical therapy, identifying opportunities for antimicrobial de-escalation and supporting antimicrobial stewardship interventions [16,43].

AI and ML algorithms may facilitate de-escalation by continuously analyzing patient characteristics, laboratory parameters, microbiological results and treatment response. By integrating these multiple sources of information, AI-based systems can identify patients who are unlikely to benefit from continued broad-spectrum antimicrobial therapy and may support more timely treatment optimization [3,44].

Several ML models have demonstrated promising results in this field. Rawson et al. developed a support vector machine (SVM) model capable of predicting the probability of co-infection or secondary bacterial infection using routinely available blood test results. The model was designed to support antimicrobial prescribing decisions and identify situations in which unnecessary antimicrobial therapy could potentially be discontinued, thereby contributing to earlier de-escalation strategies [16].

Another example was reported by Tran-The et al., who developed a ML model to identify patients requiring antimicrobial stewardship interventions. Compared with a conventional strategy based primarily on treatment duration, the model identified 41% more opportunities for antibiotic discontinuation, 16% more opportunities for intravenous-to-oral conversion, 22% more opportunities for early de-escalation and 17% more opportunities for late de-escalation. These findings suggest that ML algorithms may improve the identification of patients who would benefit from antimicrobial optimization while reducing the workload associated with manual case review [43].

Prospective clinical evidence was provided by the IDEAS study conducted by Elligsen et al. The authors integrated a predictive model with rapid antimicrobial susceptibility testing to generate individualized susceptibility probabilities and de-escalation recommendations for commonly used antibiotics. Following implementation, antimicrobial de-escalation increased from 21% to 29%, while the proportion of patients receiving the narrowest adequate antimicrobial therapy increased from 44% to 55%, without compromising treatment adequacy. These results demonstrate that predictive algorithms may support safer and more effective de-escalation strategies in routine clinical practice [45].

Taken together, these studies suggest that AI-assisted de-escalation systems may help clinicians identify opportunities for antimicrobial optimization earlier than conventional approaches. By supporting antibiotic discontinuation, narrowing antimicrobial spectrum and facilitating transition to targeted therapy, ML models have the potential to reduce unnecessary antimicrobial exposure and strengthen antimicrobial stewardship efforts. Nevertheless, further prospective multicenter studies are required to confirm their impact on clinical outcomes and to determine their applicability across different healthcare settings [1,44].

Representative studies evaluating AI and ML applications for AMR prediction, susceptibility testing, de-escalation support and surveillance are summarized in Table 2.

## 7. Implementation Challenges

### 7.1. Data Quality and Standardization

The clinical performance of AI models depends on the quality, completeness and consistency of the clinical and microbiological data used for model development and validation. In clinical microbiology, variability in specimen collection, laboratory protocols, diagnostic platforms, AST methods and reporting practices may affect dataset harmonization across institutions. Periodic updates of EUCAST and CLSI breakpoints, differences in pathogen nomenclature and heterogeneous data formats may further complicate the development of robust and generalizable models [6,9,10].

The availability of high-quality, structured and interoperable datasets is therefore essential for reliable model development and reproducibility across healthcare settings. Accurate integration of microbiological information with relevant clinical variables can improve the clinical relevance of AI predictions and facilitate external validation and implementation [1,6,9].

Federated learning may provide a potential strategy for overcoming the privacy and data-sharing barriers that limit the development of multicentre AMR prediction models. In this approach, participating institutions train models locally and share model parameters or updates rather than raw patient or microbiological data, allowing collaborative model development across geographically distributed datasets. This may improve access to more diverse populations while reducing the need for centralized data aggregation. However, federated learning does not eliminate all implementation challenges, as differences in data distributions, laboratory methods, clinical practices and local resistance epidemiology may affect model convergence and generalizability. Additional privacy-preserving measures and rigorous validation are therefore still required before federated models can be adopted in clinical practice [46,47].

### 7.2. Genotype–Phenotype Mismatch

Genotype–phenotype discordance represents an important limitation for AI models that rely primarily on genomic data to predict antimicrobial susceptibility. As discussed in Section 5.2, genomic information does not always translate directly into the phenotypic response of a microorganism to antimicrobial exposure, which may limit the clinical reliability and transferability of sequencing-based predictions [8,31].

This limitation is particularly relevant when genomic models are applied across different pathogens, resistance mechanisms or healthcare settings. Predictions based on known genetic determinants may be affected by incomplete knowledge of resistance mechanisms, variability in gene expression and difficulties in correlating genomic findings with MIC values and clinically defined susceptibility categories [8].

Consequently, genomic predictions should not be considered interchangeable with phenotypic susceptibility testing without appropriate validation. Integrating genomic information with phenotypic, microbiological and clinical data may provide a more robust basis for AI-assisted AMR prediction, while phenotypic confirmation remains important when genotype-based predictions are used to support clinical decision-making [8,31].

### 7.3. Methodological Appraisal and Clinical Evaluation

The clinical implementation of AI-based AMR prediction requires appraisal beyond the reported performance of individual models. High AUROC or accuracy alone does not establish that a model is methodologically robust, generalizable or clinically useful. The evaluation of published models should therefore consider the study design, definition of predictors and outcomes, handling of missing data and class imbalance, validation strategy, calibration, risk of bias and applicability to the intended clinical setting [48,49].

Several reporting and appraisal frameworks can support this evaluation. TRIPOD+AI provides guidance for the transparent reporting of clinical prediction models developed or evaluated using regression or machine-learning methods, whereas PROBAST+AI supports the assessment of methodological quality, risk of bias and applicability of prediction models [50,51]. When AI-based systems progress toward clinical use, early-stage live evaluation should additionally address their integration into clinical workflows, safety and real-world performance, as emphasized by the DECIDE-AI framework [52]. For AI-based diagnostic accuracy studies and prospective clinical evaluation, STARD-AI and the SPIRIT-AI and CONSORT-AI extensions provide additional guidance for reporting diagnostic studies, clinical trial protocols and completed trials involving AI interventions [49,53,54].

Class imbalance represents a particular challenge in AMR prediction because resistant organisms or specific resistance phenotypes may constitute a minority of observations. In such settings, AUROC alone may not fully characterize performance for the clinically relevant positive class. Complementary reporting of AUPRC and class-specific measures, including sensitivity, specificity, PPV and NPV, can provide a more informative assessment of model performance, particularly when the prevalence of resistance is low [49].

Discrimination should also be distinguished from calibration and clinical usefulness. A model may correctly rank patients or isolates according to their relative probability of resistance while systematically overestimating or underestimating the absolute risk. Calibration should therefore be assessed, particularly when predicted probabilities are intended to guide empirical antimicrobial therapy [48]. In addition, the appropriate decision threshold depends on the relative consequences of false-negative and false-positive predictions. Missing a resistant organism may result in initially ineffective antimicrobial therapy, whereas an unnecessary prediction of resistance may promote avoidable use of broad-spectrum agents. Decision-curve analysis and net-benefit approaches may therefore provide useful complementary measures by evaluating whether model-guided decisions offer clinical benefit across clinically relevant probability thresholds [48,49].

### 7.4. External Validation and Model Drift

Although many AI models demonstrate excellent performance during internal validation, their predictive accuracy may decline when applied to different healthcare settings because patient characteristics, antimicrobial prescribing practices, laboratory procedures and microbial epidemiology vary across institutions. Consequently, models developed using single-centre datasets may perform less accurately in hospitals with different resistance patterns, diagnostic workflows or patient populations, limiting their generalizability [7,10].

Validation strategies may also influence reported performance. When multiple isolates or clinical episodes from the same patient are included, random isolate-level splitting may place highly similar observations across training and test sets, potentially leading to information leakage and overly optimistic estimates. Similarly, closely related or clonally clustered isolates may inflate apparent performance if represented across both datasets. Patient-level splitting, together with appropriate consideration of repeated isolates and clonal relatedness, can therefore provide a more realistic assessment of generalizability [55]. Temporal validation and geographical or external validation across independent institutions are further required to assess robustness to changes in patient populations, clinical practice, circulating pathogens and resistance patterns [50].

Model performance may also change after deployment as resistance mechanisms, circulating clones, antimicrobial consumption and local epidemiology evolve over time. Continuous performance monitoring and, when necessary, recalibration or model updating are therefore required to maintain reliable clinical performance [10,56,57].

### 7.5. Interoperability and Workflow Integration

The successful implementation of AI in antimicrobial stewardship depends on the effective integration of relevant clinical and microbiological information within existing healthcare workflows. Fragmented digital systems and inconsistent data structures may limit the availability of information required for reliable predictions and hinder the transfer of AI models across different healthcare settings [6,9].

Beyond technical integration, AI systems must be embedded into existing diagnostic and prescribing workflows in a manner that supports clinical practice without increasing clinician workload or disrupting established antimicrobial stewardship processes. AI-generated predictions should therefore be available at clinically relevant time points and presented through user-friendly decision-support interfaces that complement, rather than replace, clinical judgment. Effective workflow integration may facilitate earlier antimicrobial optimization and improve clinician acceptance of AI-assisted decision-making [6,9].

### 7.6. Explainability, Bias and Human-in-the-Loop Oversight

Explainability represents a key requirement for the safe implementation of AI in antimicrobial stewardship. Accurate predictions alone are insufficient for clinical adoption if clinicians cannot understand the rationale underlying AI-generated recommendations. Explainable artificial intelligence (XAI) methods, including SHAP (SHapley Additive exPlanations) and Local Interpretable Model-agnostic Explanations (LIME), improve model transparency by identifying the variables that contribute most to individual predictions. In addition, calibration plots and regular clinical audits may help evaluate model performance and detect changes over time, thereby increasing clinician confidence in AI-assisted decision-making [16,58,59].

Another important challenge is the potential introduction of bias during model development. AI models trained on datasets derived from a single institution or a limited patient population may inadvertently reflect local epidemiology, antimicrobial prescribing practices or demographic characteristics, reducing their applicability in different clinical settings. Identifying and minimizing these sources of bias is essential to improve model fairness, reliability and generalizability [1,60].

Despite continuous advances in AI, these systems should support rather than replace clinical decision-making. A human-in-the-loop approach remains essential to ensure that AI-generated recommendations are interpreted within the clinical context of each patient. The final antimicrobial treatment decision should always remain the responsibility of the treating clinician and the antimicrobial stewardship team, while AI serves as a decision-support tool that complements clinical expertise rather than replacing it [16,56].

### 7.7. Cybersecurity, Governance and Regulation

The implementation of AI in clinical microbiology requires robust cybersecurity measures and comprehensive data governance frameworks. AI models frequently rely on electronic health records, laboratory information systems, microbiological databases and, in some cases, genomic information, all of which contain sensitive patient data. Secure data storage, controlled access, anonymization procedures and regular security audits are therefore essential to protect patient privacy and ensure the safe use of AI systems in routine clinical practice [61].

Clear governance frameworks are equally important to define responsibilities for data management, model updates and regulatory compliance throughout the AI lifecycle, ensuring transparent post-deployment oversight and patient safety [1,56].

Finally, ethical and regulatory considerations remain fundamental for the responsible adoption of AI in antimicrobial stewardship. Regulatory approval, continuous validation and appropriate clinical oversight should accompany technological advances to ensure that AI systems remain safe, transparent and clinically effective. Together, cybersecurity, governance and regulatory compliance provide the foundation for the responsible integration of AI into routine microbiology laboratories and antimicrobial stewardship programs [1,56,61,62].

In the European context, AI systems intended to support diagnostic interpretation or patient-specific clinical decision-making may also require assessment within the regulatory frameworks for medical devices or in vitro diagnostic medical devices, depending on their intended purpose and functionality. The EU AI Act introduces additional requirements for certain high-risk AI systems, including risk management, data governance, human oversight and post-market monitoring, while operating alongside the MDR and IVDR frameworks. These considerations are particularly relevant for AI-based AMR prediction because model performance may change over time as local epidemiology, patient populations or laboratory practices evolve. In this context, regulatory approaches such as the FDA Predetermined Change Control Plan (PCCP) framework provide a useful model for predefined, validated and controlled modifications throughout the lifecycle of AI-enabled medical software [63,64].

General-purpose large language models may assist with the synthesis and interpretation of microbiological or clinical information, but their use in patient-specific antimicrobial decision-making raises concerns regarding data privacy, cybersecurity, reproducibility and variable model outputs. Their clinical use should therefore require appropriate data governance, task-specific validation and clinician oversight [65].

## 8. Practical Roadmap for Implementation

The implementation of AI in antimicrobial stewardship requires appropriate technical infrastructure and the availability of reliable clinical and microbiological data. Predictive models should be integrated into routine clinical workflows in a way that supports existing antimicrobial stewardship practices and enables their use at clinically relevant decision points [6,9].

AI recommendations should support existing antimicrobial stewardship practices and remain subject to clinician and stewardship team oversight. However, final treatment decisions should remain under the supervision of the treating clinician and the antimicrobial stewardship team, following a human-in-the-loop approach [16,56].

Safe implementation additionally requires appropriate model evaluation, governance, cybersecurity and ongoing performance oversight. Clinician acceptance and continuous audit are also essential to ensure that AI systems provide meaningful support within routine clinical practice [56,61].

## 9. Limitations of This Review

This review has several limitations. First, the literature search was restricted to PubMed/MEDLINE and Google Scholar and did not include additional bibliographic databases such as Embase, Scopus or Web of Science. Relevant studies may therefore have been missed, introducing potential selection bias. Second, only English-language publications were considered, which may have introduced language bias. Although the search strategy was designed to identify literature across the clinical, microbiological, technological and bioengineering aspects of AI-assisted AMR prediction, the breadth of this topic and the use of a narrative review methodology may have influenced study selection and interpretation.

In addition, the review did not include a formal risk-of-bias assessment of the individual studies or a quantitative synthesis of their findings. The included studies were heterogeneous with respect to patient populations, microorganisms, data sources, AI methodologies, outcomes and validation strategies, limiting direct comparison of reported performance. Literature identification and selection were performed by a single author, which may have introduced subjective selection bias. Finally, because the literature was rapidly evolving, more recent developments may not have been captured after the final search date.

## 10. Conclusions

Current evidence suggests that AI has potential applications in patient-level resistance risk prediction, pathogen-level antimicrobial susceptibility prediction, surveillance and antimicrobial stewardship, although the level of evidence differs across these applications. Evidence for patient-level prediction and pathogen-level susceptibility prediction is derived predominantly from retrospective studies, with limited external and prospective validation. Surveillance applications remain comparatively preliminary, whereas AI-assisted antimicrobial stewardship has the most direct evidence of clinical implementation, although prospective outcome data remain scarce. Overall, the current literature demonstrates promising technical performance and feasibility rather than established clinical effectiveness. Broader implementation will require prospective clinical evaluation and demonstration of meaningful clinical and stewardship benefits.

## Figures and Tables

**Table 1:** Comparison of bioengineering platforms for AI-enabled antimicrobial resistance prediction.

Platform	Data Type Generated	Approximate Time to Result	Resistance Information	Cost/Infrastructure	Clinical Readiness
MALDI-TOF MS	Proteomic mass spectra	Minutes after isolate; culture required	Resistance-associated spectral patterns; limited mechanism resolution	High capital, low per-test cost	Routine ID; emerging for AMR prediction
WGS	Whole-genome sequence data	Days	Resistance genes, mutations and mobile elements; genotype–phenotype mismatch	High cost; sequencing + bioinformatics	Research/surveillance; selective clinical use
mNGS	Metagenomic sequence data	Hours–days	Pathogens and resistance genes; contamination and gene–pathogen attribution	High cost; sequencing + bioinformatics	Emerging; limited routine use
Automated AST	Growth/MIC and susceptibility data	16–36 h after isolate preparation	Phenotypic susceptibility; no direct molecular mechanism	Moderate–high; established platforms	Routine clinical use
Microfluidic AST	Real-time growth and phenotypic signals	~2–6 h	Rapid phenotypic susceptibility/MIC; no direct mechanism identification	Specialized platform; variable requirements	Early clinical implementation
Biosensors	Electrochemical, optical or sensor signals	Minutes–hours	Target-specific genes, biomarkers or phenotypic responses	Platform-dependent; potentially portable	Emerging; mainly validation/research

**Table 2:** Representative studies of AI applications in patient-level AMR prediction, pathogen-level susceptibility prediction and antimicrobial stewardship.

Technology Platform	Representative Study	Country/Setting	Sample Size	AI/ML Approach	Validation	Key Finding	Main Limitation
**A. Patient-level prediction**							
EHRs + microbiological database	**Alparslan et al.**	Turkey; single tertiary-care ICU	289 patients (159 CRKP; 130 controls)	XGBoost	Internal, single-center	Predicted carbapenem-resistant *Klebsiella pneumoniae* infection using clinical, laboratory and microbiological data (accuracy 83%; sensitivity 88%, AUROC 0.91), potentially supporting earlier identification before conventional AST.	Retrospective single-center study; pathogen-specific model; no external validation.
EHRs	**Ziaian et al.**	Southern Iran; surgical ICU, tertiary-care center	106 patients	XGBoost; logistic regression baseline	Five-fold internal cross-validation	Predicted the risk of MDR/XDR infection using EHR data with SHAP-based feature interpretation, supporting patient risk stratification, test ROC-AUC 0.786, mean fivefold cross-validation AUC 0.896 ± 0.05, accuracy 70%.	Small exploratory single-center study; limited generalizability.
Multi-institutional EHRs	**Kim et al.**	South Korea; three tertiary hospitals	59,551 patients	Multi-task learning; hard and soft parameter sharing	External validation	Predicted resistance to multiple antimicrobial classes using longitudinal EHR data (mean AUROC 0.796; mean AUPRC 0.803), potentially supporting individualized empirical therapy.	Performance depended on previous microbiological history; developed within a single national healthcare system.
EHRs + microbiological database	**Yuan et al.**	Oxfordshire, UK; hospital and community data	4709 infection episodes (4243 patients)	XGBoost	Temporal validation (independent test cohort)	Predicted antimicrobial resistance in Enterobacterales bloodstream infections (AUROC 0.723–0.827), potentially supporting empirical antimicrobial selection.	Moderate predictive performance; single healthcare network; requires local adaptation.
EHRs + microbiological database	**Wang et al.**	Development: MIMIC-IV, USA; external validation: three medical centers in China	1251 development + 362 external validation patients	XGBoost and seven additional ML algorithms	External multicenter validation	Predicted the risk of MDRO infection in catheter-related bloodstream infections (AUROC 0.877 development; 0.851 external validation), potentially supporting infection control and surveillance.	Restricted to catheter-related bloodstream infections; requires local validation before implementation.
**B. Pathogen-level prediction**							
MALDI-TOF MS	**Wang et al. (CRKP)**	China; single tertiary hospital	171 *K. pneumoniae* isolates (95 CRKP; 76 CSKP)	Random forest, SVM and SVM-K	Internal, single-center	Predicted carbapenem resistance directly from MALDI-TOF MS spectra (accuracy 91%, sensitivity 89%, specificity 94%, AUC 0.936), potentially enabling earlier susceptibility assessment.	Single-center study; limited to CRKP isolates; no prospective clinical validation.
MALDI-TOF MS	**Wang et al. (VREfm)**	Taiwan; two tertiary medical centers	7997 *E. faecium* isolates	1D convolutional neural network (CNN); comparison with RF and XGBoost	External validation	Predicted vancomycin resistance in *Enterococcus faecium* using MALDI-TOF MS spectra (AUROC 0.887).	Performance varied according to sequence type distribution; limited to one pathogen.
MALDI-TOF MS	**Wang et al. (hVISA)**	Taiwan; single tertiary hospital	125 MRSA isolates (35 hVISA/VISA; 90 VSSA)	SVM, random forest, k-nearest neighbors and decision tree	Nested cross-validation	Predicted heteroresistant vancomycin-intermediate *Staphylococcus aureus* (AUC 0.79, sensitivity 0.77, specificity 0.81), potentially supporting antimicrobial selection.	Small dataset; no prospective validation; moderate predictive performance.
mNGS	**Li et al.**	China;	113 culture-confirmed isolates for performance evaluation; 114 patients for hypothetical clinical impact	Machine-learning model using mNGS sequencing data	Internal validation	Predicted antimicrobial susceptibility from metagenomic sequencing data (accuracy 93.8%) with shorter turnaround time than conventional methods, potentially facilitating earlier therapy optimization.	No prospective clinical evaluation; reduced performance for *P. aeruginosa*; high cost and limited availability of mNGS.
**C. AI-assisted antimicrobial stewardship**							
AI-assisted antimicrobial stewardship	**Elligsen et al. (IDEAS)**	Canada; large academic tertiary-care hospital	182 pre-intervention and 201 post-intervention bacteremia episodes	Multivariable susceptibility prediction models integrated with existing resistance-prediction methods	Prospective clinical implementation	De-escalation increased from 21% to 29%; use of the narrowest adequate therapy increased from 44% to 55%, without compromising treatment adequacy	Single-center implementation; broader external validation and assessment of generalizability are required
EHRs + antimicrobial stewardship database	**Tran-The et al.**	Republic of Korea; tertiary academic hospital	19,468 patients; 306,193 hospitalized days for the largest cohort	XGBoost and LightGBM; SHAP	Hold-out test validation	Identified additional opportunities for antimicrobial discontinuation, de-escalation and IV-to-oral conversion using AI-assisted stewardship.	Single-center implementation; clinical outcomes not prospectively evaluated; recommendations required clinician validation.

## Data Availability

No new data were created or analyzed in this study. Data sharing is not applicable to this article.

## Supplementary Materials

The following supporting information can be downloaded at: https://www.mdpi.com/article/10.3390/medicina62091651/s1, Table S1. Principal database-specific literature search strategies used across the predefined thematic domains of the narrative review.

## Abbreviations

AI	Artificial intelligence
AMR	Antimicrobial resistance
AST	Antimicrobial susceptibility testing
AUC	Area under the curve
AUPRC	Area under the precision–recall curve
AUROC	Area under the receiver operating characteristic curve
CDC	Centers for Disease Control and Prevention
CRKP	Carbapenem-resistant *Klebsiella pneumoniae*
DECIDE-AI	Developmental and Exploratory Clinical Investigations of Decision-support systems driven by Artificial Intelligence
EARS-Net	European Antimicrobial Resistance Surveillance Network
ECDC	European Centre for Disease Prevention and Control
EHR	Electronic health record
ESKAPEE	*Enterococcus faecium*, *Staphylococcus aureus*, *Klebsiella pneumoniae*, *Acinetobacter baumannii*, *Pseudomonas aeruginosa*, *Enterobacter* spp., and *Escherichia coli*
FDA	Food and Drug Administration
GLASS	Global Antimicrobial Resistance and Use Surveillance System
hVISA	Heterogeneous vancomycin-intermediate *Staphylococcus aureus*
ICU	Intensive care unit
IDEAS	Improving Decision Making in Empiric Antibiotic Selection
IVDR	In Vitro Diagnostic Medical Devices Regulation
LightGBM	Light Gradient Boosting Machine
LIS	Laboratory information system
MALDI-TOF MS	Matrix-assisted laser desorption/ionization time-of-flight mass spectrometry
MDR	Multidrug-resistant
MDRO	Multidrug-resistant organism
MIC	Minimum inhibitory concentration
ML	Machine learning
mNGS	Metagenomic next-generation sequencing
MRSA	Methicillin-resistant *Staphylococcus aureus*
NHSN	National Healthcare Safety Network
NPV	Negative predictive value
PCCP	Predetermined Change Control Plan
PPV	Positive predictive value
PROBAST+AI	Prediction model Risk Of Bias ASsessment Tool for Artificial Intelligence
SHAP	Shapley Additive Explanations
SPIRIT-AI	Standard Protocol Items: Recommendations for Interventional Trials–Artificial Intelligence
STARD-AI	Standards for Reporting Diagnostic Accuracy Studies–Artificial Intelligence
SVM	Support vector machine
TRIPOD+AI	Transparent Reporting of a multivariable prediction model for Individual Prognosis Or Diagnosis–Artificial Intelligence
VISA	Vancomycin-intermediate *Staphylococcus aureus*
VREfm	Vancomycin-resistant *Enterococcus faecium*
VSSA	Vancomycin-susceptible *Staphylococcus aureus*
WGS	Whole-genome sequencing
WHO	World Health Organization
XDR	Extensively drug-resistant
XGBoost	Extreme Gradient Boosting
