# Supplementary material for: Artificial Intelligence and Bioengineering Approaches for Antimicrobial Resistance Prediction
Source: Medicina (Kaunas). 2026 Aug 28;62(9):1651. doi: 10.3390/medicina62091651 (PMC13608866; doi:10.3390/medicina62091651)
Supplement: Supplementary file 1 [file medicina-62-01651-s001.zip › medicina-4520127-supplementary.pdf]

Thematic domain	Representative PubMed/MEDLINE search strategy	Representative Google Scholar search strategy
**AI and AMR prediction**	("artificial intelligence" OR "machine learning" OR "deep learning") AND ("antimicrobial resistance" OR "antibiotic resistance" OR "drug resistance") AND (prediction OR predictive)	"artificial intelligence" antimicrobial resistance prediction
**Patient-level resistance prediction and clinical data**	("machine learning" OR "artificial intelligence") AND ("antimicrobial resistance" OR multidrug-resistant OR MDR) AND ("electronic health records" OR clinical data OR patient data)	"machine learning" antimicrobial resistance electronic health records prediction
**AI-assisted antimicrobial stewardship**	("artificial intelligence" OR "machine learning") AND ("antimicrobial stewardship" OR antibiotic stewardship) AND ("clinical decision support" OR prescribing OR de-escalation)	"artificial intelligence" antimicrobial stewardship clinical decision support
**Whole-genome sequencing and AI**	("whole genome sequencing" OR WGS OR genomics) AND ("machine learning" OR "artificial intelligence") AND ("antimicrobial resistance" OR antibiotic resistance)	"whole genome sequencing" machine learning antimicrobial resistance prediction
**Metagenomic sequencing and AI-assisted susceptibility prediction**	("metagenomic next-generation sequencing" OR mNGS OR metagenomics) AND ("machine learning" OR "artificial intelligence") AND ("antimicrobial susceptibility" OR resistance prediction)	mNGS machine learning antimicrobial susceptibility prediction
**MALDI-TOF MS and machine-learning-based resistance prediction**	("MALDI-TOF" OR "mass spectrometry") AND ("machine learning" OR "deep learning" OR artificial intelligence) AND ("antimicrobial resistance" OR susceptibility)	"MALDI-TOF" machine learning antimicrobial resistance
**Automated and microfluidic AST**	("automated antimicrobial susceptibility testing" OR "rapid susceptibility testing" OR microfluidic*) AND ("antimicrobial susceptibility" OR AST) AND (artificial intelligence OR "machine learning" OR digital)	microfluidic antimicrobial susceptibility testing machine learning
**Biosensors and AMR detection**	(biosensor* OR "electrochemical sensor*" OR "optical sensor*") AND ("antimicrobial resistance" OR resistance genes OR "antimicrobial susceptibility")	biosensors antimicrobial resistance detection
**Digital microbiology and clinical decision support**	("digital microbiology" OR "clinical decision support" OR "electronic health records") AND ("artificial intelligence" OR "machine learning") AND (microbiology OR antimicrobial)	"digital microbiology" artificial intelligence clinical decision support
**Methodological evaluation of AI-based clinical prediction models**	("clinical prediction model*" OR "machine learning model*") AND (validation OR calibration OR discrimination OR "risk of bias" OR "clinical utility")	AI clinical prediction models validation calibration clinical utility
**Class imbalance and performance metrics**	("machine learning" OR "clinical prediction") AND ("class imbalance" OR imbalanced) AND (AUROC OR AUPRC OR "precision recall")	"class imbalance" AUROC AUPRC clinical prediction models
**AI implementation, governance and regulation**	("artificial intelligence" OR "AI-enabled") AND (healthcare OR clinical) AND (governance OR regulation OR cybersecurity OR "model drift" OR monitoring)	AI healthcare regulation model drift governance

Thematic domain	Representative PubMed/MEDLINE search strategy	Representative Google Scholar search strategy
**Federated learning and healthcare data**	("federated learning") AND (healthcare OR clinical OR hospital) AND ("machine learning" OR artificial intelligence)	"federated learning" healthcare artificial intelligence

Abbreviations: AI, artificial intelligence; AMR, antimicrobial resistance; AST, antimicrobial susceptibility testing; AUROC, area under the receiver operating characteristic curve; AUPRC, area under the precision–recall curve; EHR, electronic health record; MALDI-TOF MS, matrix-assisted laser desorption/ionization time-of-flight mass spectrometry; mNGS, metagenomic next-generation sequencing; WGS, whole-genome sequencing.

Supplementary Table S1. Principal database-specific literature search strategies used across the predefined thematic domains of the narrative review.
